# Abortion Trajectory, Timing, and Access Study (ATTAS): study protocol

**DOI:** 10.1186/s13690-024-01418-x

**Published:** 2024-11-13

**Authors:** Anna Wallays, Sarah Van de Velde

**Affiliations:** University of Antwerp, Antwerp, Belgium

**Keywords:** Abortion, Trajectory, Barrier, Legal limit, Delay, Flanders

## Abstract

**Background:**

This study protocol outlines the phased construction of a questionnaire, data collection, and a strategy for analysis within the framework of the ATTAS project. This study has two primary objectives. First, it allows us to map the duration of the various phases of the abortion trajectory for women presenting for abortion in Flanders, Belgium. Second, it identifies barriers that cause delays within these phases.

**Methods:**

The questionnaire was distributed to all women seeking abortion care at one of the five Flemish abortion centers; specifically, the LUNA centers, which are located in Ostend, Ghent, Antwerp, and Hasselt, as is the VUB-Dilemma center in Brussels during the fall and winter of 2023–2024. Ethical clearance for the described research was obtained from the University of Antwerp’s Ethics Committee for the Social Sciences and Humanities (reference numbers: SHW_2023_48_1 and SHW_2023_48_2).

**Discussion:**

The collected data provide a dataset on the abortion trajectories of Flanders women who presented for abortion. This study protocol outlines the ATTAS project’s rationale, phased development, and implementation of the questionnaire, as well as the upcoming data analyses. To our knowledge, this is the first study within the Flemish context to investigate abortion trajectories, timing, and access. Furthermore, this study protocol provides a phased and systematic approach to adapt validated research instruments to fit within diverse legal and cultural contexts. Building on this protocol, future research will seek to advance reproductive justice for all women in Belgium.

**Supplementary Information:**

The online version contains supplementary material available at 10.1186/s13690-024-01418-x.

**Table:** 

Text box 1 Contributions to the literature
• There is limited evidence on how to combine and adapt existing research instruments for mapping abortion trajectories and identifying barriers to abortion care in the Belgian context.
• This protocol gives a detailed description of the phased development and implementation of the questionnaire for mapping abortion trajectories and identifying barriers.
• The data collection will enable an in-depth understanding of abortion trajectories among women who seek abortion care.

## Background

In Belgium, there has been a noticeable trend toward abortion at less advanced gestational ages in recent decades [1, 2]. However, patient records from abortion centers show that women present for abortion at a wide range of gestational ages [LUNA Patient Records - unpublished observations], ranging from three weeks to the full term. In Belgium, abortion on request is legally available up to 14 weeks of gestation. In Flanders, the Dutch-speaking northern half of Belgium, where the current study is based and where more than half of the Belgian population lives – between 120 and 170 women present each year beyond this legal limit of 14 weeks [LUNA Patient Records - unpublished observations]. Women seeking abortions beyond this legal limit have to seek care in neighboring countries. The latter creates additional emotional, practical, and financial barriers for a group of women who are also more likely to be in a more vulnerable socioeconomic position [3]. Studies building on this protocol will therefore best incorporate an intersectional approach. Additionally, while abortion is generally a safe medical procedure [4, 5], this procedure carries a greater risk of complications when performed at more advanced gestational ages.

This protocol describes the ATTAS project, which aims to map the trajectories that women follow to access abortion care in the Belgian context and the barriers therein. The findings of this study are highly topical given the current political debates on the decriminalization of abortion in Belgian legislation and political proposals to extend the legal limit for abortion-related care from 14 to 18 weeks. Nevertheless, these initiatives and the surrounding political debate were postponed until the next legislative period. One of the reasons for this was that both proponents and opponents of proposed legislative changes pointed to the lack of research on the issue in the Belgian context. While the issue has been extensively studied in other contexts [6–13], research on the topic in Belgium is urgently needed.

## Concepts

### Abortion trajectory

**Fig. 1:**
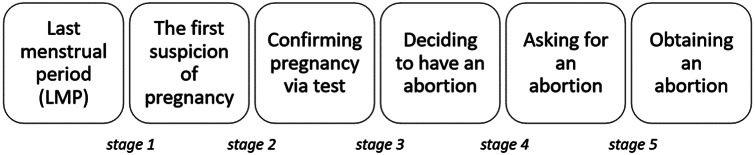
Ideal type abortion trajectory

Numerous studies investigating abortion decision-making processes, use the concept of an ‘abortion trajectory’ or its equivalents. However, there are multiple interpretations of the concept. In a study by Finer et al. [5], it was conceptualized as “the process of obtaining an abortion — from the woman’s last menstrual period (LMP) to the time she suspects she is pregnant, from suspecting pregnancy to confirming her suspicion via a positive pregnancy test, from confirming the pregnancy to deciding to have an abortion, from deciding to have an abortion to beginning to seeking abortion services and from beginning to seeking abortion services to obtaining an abortion”. It is important to note that Finer et al., like several other authors, assumed a certain ideal type of abortion trajectory [8, 11, 14]. According to this ideal type, the trajectory consists of six consecutive steps or five sequential stages (Fig. 1).

However, there are a few nuances to this ideal trajectory. First, not all women (consciously) follow every step, with some steps being skipped or revisited [15]. Second, as Coast et al. [16] note, “abortion-related care-seeking cannot be understood only through a linear course of action; it is a process that responds to changing circumstances and experiences”.

It is also crucial to recognize that the term ‘trajectory’ inherently conveys the concept of time. This understanding is pivotal when considering abortion care-seeking, as the possibility of legal abortion diminishes as pregnancy progresses, with the exact limit depending on the context. The assumptions about abortion trajectories and the barriers identified in previous studies are strongly influenced by their specific contexts. Most studies that have tracked the duration of various stages were, to date, conducted in the US or in Great Britain [5, 6, 9, 10]. This makes it especially pertinent to explore these issues in the context of Flanders in Belgium.

Apart from the 14-week legal limit, Belgian Law stipulates other conditions that must be fulfilled to access abortion. For instance, women are required to schedule an appointment for a first consultation at the abortion clinic [17]. During this session with a healthcare worker (HCW), the patients received counseling and underwent a medical examination. Following this first consultation, the compulsory six-day waiting period between the counseling session and the abortion treatment commenced. As of October 2018, should women approach the end of the 14-week limit, these six days can be added to the 14-week limit. This mandatory waiting period will therefore ensure that the fifth stage in the abortion trajectory in the Belgian context—from the initial abortion request to the procedure itself— lasts a minimum of seven days. It is essential to comprehend how abortion trajectories in this context are structured and acquired over a certain duration.

### Barrier(s)

Extensive research underscores that women face numerous barriers throughout their abortion trajectory that influence the duration of the different stages [6–13] (Supplementary material - Table 1). Here, ‘barriers’ are understood as factors that potentially cause delay(s) or complexity in the abortion trajectory. They can occur in any abortion trajectory, can manifest at any stage, and can vary in nature, being practical, moral, or situational. Each stage of the abortion trajectory has its specific barriers that can cause delays. In some cases, these delays culminate in women seeking abortions beyond the legal limit for abortion, which, according to Belgian law, is 14 weeks of gestational age for elective abortions. However, it is important to highlight that in addition to these barriers, there are also facilitators that can accelerate the path to timely abortion care. Available research, however, indicated that facilitators mainly function on a broader scale, often through policy structures and the organization of healthcare services e.g. provider availability or costs of the treatment [18]. Consequently, we argue that the absence of specific barriers, whether practical, moral, or situational, may inherently act as facilitators.

### Research objectives

The overall objective of this study was to explore the possible steps involved in, and the reasons for delays in obtaining abortion-related care in the Belgian context.

- **Objective 1 (RO1)**: To map the duration of the different phases of the abortion trajectory of women presenting for abortion in Belgium.
- **Objective 2 (RO2)**: To identify possible barriers faced by women in their abortion trajectory in the Belgian context.
- **Objective 3 (RO3)**: To compare the abortion trajectories of women presenting within and beyond the legal limit in the Belgian context, thereby assessing both within and between-group differences.
- **Objective 4 (RO4)**: To compare the barriers faced by women presenting for abortion within and beyond the legal limit in the Belgian context, again assessing both within and between-group differences.

## Methods & development of the questionnaire

### Operationalization of the abortion trajectory

The current study builds upon the abortion trajectory inventory developed by Finer et al. [5]. Data on this abortion trajectory is collected through a self-administered questionnaire in the form of a calendar that surveys the timing of steps in the process of obtaining an abortion. Event history calendars (EHCs) have been demonstrated to be effective tools for collecting retrospective data in other studies [19, 20]. In addition, the EHC method has been used in SRHR research and other surveys that address sensitive topics [21–23]. As in previous research, we rely on the ideal type of abortion trajectory. However, we do not assume a certain chronological order in these steps, since the dates surveyed can be indicated freely. We assumed that an abortion trajectory comprises different, not necessarily chronological, steps, resulting in multiple, sometimes overlapping stages. For pragmatic reasons, we only measure when a respondent enters a particular stage for the first time; the respondent can indicate this date on the calendar integrated into the online questionnaire. The following key dates are surveyed explicitly: the first day of LMP, the day on which one first suspected pregnancy, and the day on which a (first) positive pregnancy test was taken.

Since we want to minimize possible recall bias even more and avoid double-questioning our respondents, we do not survey the dates of other steps in the trajectory directly. The five participating centers already record these data in their patient records. We link these data to our survey data, allowing us to reconstruct the entire trajectory without double querying. The day on which the abortion decision is made can be hard to recall since it is often not a clear-cut moment in time. For this key date, we, therefore, rely on the day the abortion center was contacted (for the first time), and the appointment for the first consultation was made. In general, during the first consultation, the appointment for the abortion itself is made. Accordingly, the date of the completion of the questionnaire, which is equivalent to the first consultation, serves as a proxy for the day on which an abortion is first requested. The last key date, the abortion procedure, is recorded by the centers themselves and is added to our dataset through the link between the center’s databases and our survey data. This link is established based on the respondents’ date of birth, which is recorded in the patient record at the center and which respondents are asked to provide at the beginning of the survey. In rare cases where two or more respondents share the same date of birth, we can still accurately link survey responses to patient records based on the day the survey is completed. This link also provides access to the respondents’ background data, such as parity, relationship status, and education level.

### Operationalization of ‘barriers’

#### Literature review

To operationalize the concept of ‘barrier’, we applied a systematic approach based on an extensive literature review as a first step. Through a thorough reading of the articles cited above, we could compile a comprehensive list of possible barriers and reasons for delay. To keep an overview, the barriers were grouped by the stage of the abortion trajectory in which they are most likely to occur [6] (Supplementary material - Table 1). Because the questionnaires are completed at the time of the first consultation, barriers can only be surveyed up to this point in the abortion trajectory. Therefore, we were not able to survey possible barriers faced after presenting for abortion. Since our study focuses on the gestational age at which women present for abortion, this is not a limitation of our study.

#### Fieldwork in abortion clinics

In each of the participating abortion centers, one day of fieldwork was conducted. In this way, insight could be gained into the actual operation and daily practice of Flemish abortion centers. As a result, barriers that were identified through this insight could be added to the list – drawn up during the literature review of possible barriers (see infra) – and some barriers could be deleted that turned out not to be applicable in the Belgian context (Supplementary material - Table 1). Being present in the abortion centers for a day was also important for building contact with the center managers and HCWs. In this way, we also ensured willingness to cooperate in the next phases of adaptation and validation and the implementation of the questionnaire.

#### Focus group discussion with HCWs

Based on the theoretical knowledge gathered through the literature review, the practical knowledge gained through fieldwork, the content, and the script for the Focus Group Discussion (FGD) with HCWs could be shaped. The list of barriers compiled and edited through the literature review and fieldwork served as the starting point for this FGD. We conducted an online FGD in which eight HCWs and two researchers were present. To encourage participation in the online setting, we used PollEverywhere to obtain input from our respondents in a simple and accessible way.

The FGD started with a short explanation of the project and the goal of the FGD. We started by asking, in an open-ended way, what they think are possible barriers or factors causing delays in presenting for abortion. The list of possible barriers was subsequently displayed per stage in the trajectory. The HCWs were asked which of the displayed barriers they feel do not apply in the Flemish context, which are the most important barriers, and what barriers they have already experienced in practice that are not yet listed.

The FGD revealed some interesting results; the HCWs of the different centers agreed clearly on which barriers do not occur in the Flemish context and which are the most relevant ones. The role of context as a critical shaping factor was noted, but it was stressed that the impact should certainly not be underestimated. The HCWs indicated that some barriers that were extracted directly from the literature are not that clearly or ambiguously worded. They advised on how they would clarify or simplify them. Some listed barriers were found to be similar, so they could better be grouped. Other barriers were found to be examples of higher-level barriers and thus fit better as a subcategory. It was also noted that some barriers may occur at multiple steps in the trajectory, whether in a slightly different form or not. Furthermore, HCWs added some specific barriers to the already compiled list, such as “It dawned on me that continuing the pregnancy would not be without risk (both for health; socially and emotionally)” or “I felt ashamed that I was thinking about opting for an abortion”. With the results of this focus group in mind, the list of barriers for each step in the abortion process was thoroughly reworked by the researchers, who created items that could be included in the questionnaire (Supplementary material - Table 1).

#### Piloting questionnaire

We conducted a three-way pilot study. First, we had other researchers experienced in the field or in developing questionnaires who critically reviewed the questionnaire. They were asked to mainly pay attention to the flow of the questionnaire and the completeness and wording of the questions. Therefore, it was also decided that since the ‘asking for an abortion’ step (stage 4) had quite a long list of barriers, which are also clearly of different nature, to split it into two parts. The barriers associated with making an appointment in an abortion center are displayed first, followed by the barriers involved in physically getting to the center for the first consultation.

Second, we conducted a pilot study with women who had an abortion in the preceding year. The participants were first asked to complete the draft version of the survey on their smartphones. Some questions were displayed in different ways to ascertain which was the most accessible. Later, the researcher asked to comment on various aspects of the questionnaire, including length, content, readability, and clarity. The goal of this pilot was to ensure that every woman felt that the questionnaire captured her particular situation. Accordingly, a group of respondents was selected for this pilot consisting of four women between 23 and 37 years old. They commented extensively on which questions they felt were unclear or triggering and whether they felt they could tell their story by completing the questionnaire. Adaptations were made accordingly (Supplementary material - Table 1).

The respondents of the pilot also commented on the suggested approaches for recruiting women into the study. The participants were unanimous that it would be effective if they were personally asked to complete the questionnaire rather than through other recruitment methods (e.g., through the display of a QR code in the waiting room). Keeping the comments from the pilot in mind, we chose to work with tablets. This ensures that the request to participate in the study is in person and, at the same time, makes the completion itself very accessible.

Third, the draft of the questionnaire was sent to the coordinators of each of the abortion centers. They mainly checked whether the questions would be straightforward to interpret and answer. These three steps of piloting resulted in the final questionnaire.

#### Final questionnaire

The final questionnaire was administered online on the Qualtrics Survey Platform, a commonly used online survey platform that conforms to the EU General Data Protection Regulation (GDPR) guidelines. When accessing the questionnaire, a short informed consent is displayed first (Supplementary material – final questionnaire). Participation in the questionnaire is entirely voluntary and can be stopped at any time, and the data will be processed anonymously. A link to a more comprehensive version of the informed consent is provided for those who want more information. Once the respondent agrees to the informed consent, the actual survey starts.

In the first module of the questionnaire, three key dates in the abortion trajectory are queried (Table 1). The respondents are first asked if they remember the date of a particular step. They can indicate one of the following responses: ‘Yes, I know the exact date’, ‘No, I don’t know the date exactly, but I have an idea of when it was approximately.’ or ‘No, I don’t know’. If they indicate the first or second option, a calendar will be displayed in which they can easily indicate the exact date, on which the step queried, took place. Some steps have additional questions if a certain response is indicated (Supplementary material – final questionnaire).

**Table 1:** The modules of the questionnaire and the number of items per module

Module	Topic	Measurements	Number of items
			(+ filter questions)
*0*	*Informed consent and sociodemographic information*	*Agreement to participate*	*2*
		*Date of birth*	
1	Abortion trajectory	LMP; first suspicion of pregnancy; first positive pregnancy test	6 (+ 2)
2	Barriers	Suspecting/recognizing pregnancy; taking pregnancy test; certainty decision; difficulty decision; deciding on abortion; making appointments; getting to the abortion center	12 (+ 4)
3	Stigma and values	ILAS; knowing someone who had an abortion; EVS	6
*4*	*Further research*	*Email-address*	*1*
**Total**			**27 (+ 6)**

The second module of the questionnaire focuses on the barriers experienced in these moments (Table 1). The respondents are asked to indicate for each barrier if they felt it applied by indicating ‘Yes’ or ‘No’. For some barriers, additional questions are asked when the respondent indicates that this barrier applies to her particular situation. First, the barriers associated with suspecting pregnancy are displayed, followed by those that might occur when taking a pregnancy test. The barriers associated with the third stage, the decision-making stage, are preceded by two slider questions. On an 11-point scale, running from 0 (‘I am still very much in doubt’) to 10 (‘my decision is firm’), the extent to which the decision for abortion is already certain should be indicated. Additionally, a second 11-point scale ranging from 0 (‘not difficult at all’) to 10 (‘very difficult’) is used to indicate how difficult the decision was. The answer to this question creates a context for the coming questions and is also very useful later in interpreting the responses.

The third and final module of the questionnaire consists of items copied and adapted from existing and broadly used scales and surveys (Supplementary material - Table 1). As indicated before, we used several subscales of the ILAS scale. The ILAS scale is a validated and reliable research instrument developed by Cockrill et al. [24] that can be used in research examining abortion stigma and related outcomes (e.g., women’s health, relationships, and behavior). Given that this scale was designed to be completed after the abortion, while we queried the respondents before the procedure, some changes had to be made. In addition to the ILAS scale items, we also included two questions from the European Value Survey (EVS) on religiosity and political orientation. By including these questions, we gain a basic insight into the respondents’ contexts, which is needed to frame perceived barriers and stigma [25].

The final questionnaire consists of 27 to 33 questions, depending on which answer options are indicated (Table 1).

### Translation

The newly developed questionnaire is crafted in Dutch, the dominant language of the research team and the research setting. These questions are translated into French and English. Data from abortion centers show that the majority of the patient population (99.8%) is sufficiently proficient in one of these languages. For the remaining 0.02%, a telephone translator was consulted. The translation of the questionnaire was performed through a committee approach, ensuring that the process remained steep in the target language and avoiding the loss of quality associated with back-translation [26]. The same translation method was used to translate the ILAS scale from English into Dutch and French, while the questions obtained from the EVS were already translated into the three research languages by the EVS itself.

## Research Design

### Sampling and recruitment methods

The research population consists of all women presenting for abortion care at any of the Dutch-speaking abortion centers (LUNA Ostend, Ghent, Antwerp, and Hasselt and VUB-Dilemma in Brussels) in Belgium, regardless of age, place of residence, nationality, or any other sociodemographic characteristic. To participate in the survey, women need to be sufficiently proficient in reading and understanding one of the three survey languages (Dutch, French, English).

Respondents are recruited directly through abortion centers when they find themselves in the center for their first consultation. The recruitment of women presenting for abortion is done through HCWs working at abortion centers. All HCWs were trained on the purpose and relevance of the survey and on ways to effectively recruit women into the study. The research team also walked through the questionnaire with the HCWs so that the HCWs understood the questionnaire well and would be able to offer help with any questions that respondents may have.

After the first consultation, the HCWs provide each woman a brief explanation of the study both orally and through a printed document and ask them to complete the questionnaire. Respondents will then be able to fill out the survey either on a tablet that is offered to them by the HCW or by scanning a QR code that enables them to fill out the survey on their smartphone. We assume that the first consultation will develop a more trusting relationship between the HCW and the woman, which may also increase the successful recruitment of the woman into the study (in comparison to timing the recruitment prior to this initial intake). If a woman initially refuses to participate, the HCW will provide additional encouragement and will answer possible questions about the study and/or the questionnaire. This will be particularly the case for women who present beyond the legal limit for abortion. The same recruitment efforts will additionally be made to attract the latter group of women to the study when they present for a post-abortion medical check-up after receiving abortion care abroad.

Since the number of women who present beyond the legal limit is relatively small, there is no fixed duration of the recruitment phase. Nonetheless the data collection will continue until a sufficiently large sample size is reached. Based on the sample size estimation as detailed by Charan & Biswas [27], we intend to draw a study sample of at least 360 women from the total patient population. The data is closely monitored through the phase of data collection by comparing the collected data with the patient record data on a monthly basis. This allows us to monitor whether over- or underrepresentation by certain background characteristics (e.g., age, education level, and employment status) is present. In this way, we can map the degree of nonresponse. If necessary, additional targeted efforts will be made to obtain a representative sample.

### Ethical considerations

Several ethical considerations were taken into account in this study. Ethical approval for the focus group, the pilot, and the questionnaire was obtained from the University of Antwerp’s Ethics Committee for the Social Sciences and Humanities. A data management plan was also drawn up and approved. A risk analysis was carried out internally to ensure the privacy of the respondents. Given the importance of the timing of the questionnaire for the decision-making process, we deliberately kept the questionnaire as short as possible to avoid placing unnecessary burdens on the respondents. In addition, the questionnaire does not contain overly complex or triggering questions. We also ensured that participating in the questionnaire would not interfere with the woman’s decision-making process.

### Plan of data analysis

Following a thorough round of data cleaning and coding of missing values, descriptive analyses will be conducted to examine RO1 and RO2. The statistical software SPSS will be used to conduct all of the statistical analyses. First, the abortion trajectories will be constructed using the collected data, including a description of the specific steps within this trajectory. The data additionally allows us to analyze the frequency of possible barriers faced during the subsequent steps of the trajectory. The barriers experienced will be related to sociodemographic characteristics and the stigma and values variables. Second, conducting descriptive analyses will allow us to make statements about the average length of abortion trajectories, the minimal and maximum length, standard deviations, and variance.

To explore RO3 and RO4, we distinguish between women who applied before and those who applied beyond the legal limit. This allows us to compare the descriptive statistics for these two groups. In addition, we will examine whether there are significant differences between the barriers experienced causing delay(s) between women who present within and beyond the legal limit within the Belgian context and whether certain groups of women are more at risk for this delay.

## Discussion

This study protocol describes the rationale of the research project, in addition to the phased development and implementation of the questionnaire. The questionnaire maps the duration of the different stages in an abortion trajectory. Moreover, barriers experienced during this trajectory are also questioned. The collected data will provide a dataset on the abortion trajectories of women presenting for abortion in Flanders. The analyses that will be conducted based on the collected data are also discussed in this article. The findings from this study will serve as a valuable foundation in Belgian abortion research and thus also contribute to informing abortion-related policy and organizing practices [28]. The results of the different analyses, which will be conducted based on the collected data, will give rise to the writing of several papers. These papers will be published in peer-reviewed journals.

To our knowledge, this is the first study within the Flemish context to investigate abortion trajectories, timing, and access. Furthermore, this study protocol provides a phased and systematic approach for adapting validated research instruments to a different legal and cultural context. Researchers from other countries who also seek to map abortion trajectories and experienced barriers in their countries can go through the steps described above. In this way, they will arrive at a research tool adapted to their particular legal and cultural context surrounding abortion.

## Electronic supplementary material

Below is the link to the electronic supplementary material.

Supplementary Material 1

Supplementary Material 2

## Data Availability

No datasets were generated or analysed during the current study.

## Abbreviations

ATTAS: Abortion Trajectory, Timing, and Access Study
BOF: Bijzonder onderzoeksfonds
EHC: Event history calendar
EVS: European Values Study
GDPR: General Data Protection Regulation
HCW: Healthcare worker
ILAS: Individual Level Abortion Stigma
LMP: Last menstrual period
RO: Research objective
VUB: Vrije Universiteit Brussel

## References

[1] National Evaluation Committee on Termination of Pregnancy. Report for the benefit of Parliament January 1st 2020 - December 31th 2021. 2023.

[2] Kortsmit K, Nguyen AT, Mandel MG, Clark E, Hollier LM, Rodenhizer J, et al. Abortion Surveillance—United States 2020 MMWR Surveillance Summaries. 2022;71(10):1–27. 10.15585/mmwr.ss7110a1 PMC9707346 36417304

[3] Van de Eekert VS, Van Assche N, Sommerland K, Wouters N. Characteristics of women who present for abortion beyond the legal limit in Flanders, Belgium. Perspect Sex Reproductive Health Health. 2019;51(3):175–83. 10.1363/psrh.12116 31509652

[4] WHO. Abortion care guideline 2022 [ https://www.who.int/publications/i/item/9789240039483

[5] Finer LB, Frohwirth LF, Dauphinee LA, Singh S, Moore AM. Timing of steps and reasons for delays in obtaining abortions in the United States. Contraception. 2006;74(4):334–44. 16982236 10.1016/j.contraception.2006.04.010

[6] Lee E, Ingham R. Why do women present late for induced abortion? Best Pract Res Clin Obstet Gynaecol. 2010;24(4):479–89. 20338814 10.1016/j.bpobgyn.2010.02.005

[7] DePiñeres T, Raifman S, Mora M, Villarreal C, Foster DG, Gerdts C. I felt the world crash down on me’: women’s experiences being denied legal abortion in Colombia. Reprod Health. 2017;14(1):133. 29058629 10.1186/s12978-017-0391-5 PMC5651606

[8] Ingham R, Lee E, Clements SJ, Stone N. Reasons for second trimester abortions in England and Wales. Reprod Health Matters. 2008;16(31Suppl):18–29. 18772080 10.1016/S0968-8080(08)31375-5

[9] Foster DG, Gould H, Biggs MA. Timing of pregnancy discovery among women seeking abortion. Contraception. 2021;104(6):642–7. 34363842 10.1016/j.contraception.2021.07.110

[10] Foster DG, Jackson RA, Cosby K, Weitz TA, Darney PD, Drey EA. Predictors of delay in each step leading to an abortion. Contraception. 2008;77(4):289–93. 18342653 10.1016/j.contraception.2007.10.010

[11] Drey EA, Foster DG, Jackson RA, Lee SJ, Cardenas LH, Darney PD. Risk factors associated with presenting for abortion in the second trimester. Obstet Gynecol. 2006;107(1):128–35. 16394050 10.1097/01.AOG.0000189095.32382.d0

[12] Harries J, Orner P, Gabriel M, Mitchell E. Delays in seeking an abortion until the second trimester: a qualitative study in South Africa. Reproductive Health. 2007;4(1). 10.1186/1742-4755-4-7 PMC2039724 17883835

[13] Purcell C, Brown A, Melville C, McDaid LM. Women’s embodied experiences of second trimester medical abortion. Feminism Psychol. 2017;27(2):163–85. 10.1177/0959353517692606 PMC5431358 28546655

[14] Purcell C, Cameron S, Caird L, Flett G, Laird G, Melville C et al. Access to and experience of later abortion: accounts from women in Scotland. Perspect Sex Reprod Health. 2014;46(2). 10.1363/46e1214 24785904

[15] Marecek J, Macleod C, Hoggart L. Abortion in legal, social, and healthcare contexts. Feminism Psychol. 2017;27(1):4–14.

[16] Coast E, Norris AH, Moore AM, Freeman E. Trajectories of women’s abortion-related care: a conceptual framework. Soc Sci Med. 2018;200:199–210. 29421467 10.1016/j.socscimed.2018.01.035

[17] De Kort L. The social profile of women requesting abortion care in Flanders, Belgium: an analysis of subsequent abortions and of abortion care during the first wave of the COVID-19 pandemic. University of Antwerp; 2022.

[18] Doran F, Nancarrow S. Barriers and facilitators of access to first-trimester abortion services for women in the developed world: a systematic review. J Family Plann Reproductive Health Care. 2015;41(3):170–80. 10.1136/jfprhc-2013-100862 26106103

[19] Berchtold A, Wicht B, Surís J-C, Morselli D. Consistency of data collected through online life history calendars. Longitud Life Course Stud. 2022;13(1):145–68. 10.1332/175795921X16209324334818 35920624

[20] Morselli D, Le Goff J-M, Gauthier J-A. Self-administered event history calendars: a possibility for surveys? Contemp Social Sci. 2019;14(3–4):423–46.

[21] Becker S, Sosa D. An experiment using a Month-by-Month calendar in a Family Planning Survey in Costa Rica. Stud Fam Plann. 1992;23(6):386–91. 1293862

[22] Morselli D, Berchtold A, Suris Granell J-C, Berchtold A. On-line life history calendar and sensitive topics: a pilot study. Comput Hum Behav. 2016;58:141–9.

[23] Yoshihama M, Gillespie B, Hammock AC, Belli RF, Tolman RM. Does the life history calendar method facilitate the recall of intimate partner violence? Comparison of two methods of data collection. Social Work Res. 2005;29(3):151–63.

[24] Cockrill K, Biggs A. Can stories reduce abortion stigma? Findings from a longitudinal cohort study. Cult Health Sexuality. 2018;20(3):335–50. 10.1080/13691058.2017.1346202 28705119

[25] EVS/WVS, Joint EVSWVS. 2017–2022 Dataset GESIS, Cologne. ZA7505 Data file Version 4.0.0, 10.4232/1.14023; 2022.

[26] Fitzgerald R, Jowell R. Measurement equivalence in comparative surveys: the European Social Survey (ESS)—from design to implementation and beyond. Survey methods in multinational, multiregional, and multicultural contexts. 2010:485 – 95.

[27] Charan J, Biswas T. How to calculate sample size for different study designs in medical research? Indian J Psychol Med. 2013;35(2):121–6. 24049221 10.4103/0253-7176.116232 PMC3775042

[28] Kim CR, Tunçalp Ö, Ganatra B, Gülmezoglu AM, Group WM-AR. WHO multi-country survey on abortion-related morbidity and mortality in health facilities: study protocol. BMJ Global Health. 2016;1(3):e000113. 28588967 10.1136/bmjgh-2016-000113 PMC5321365

