# Supplementary material for: Abortion Trajectory, Timing, and Access Study (ATTAS): study protocol
Source: Arch Public Health. 2024 Nov 13;82:211. doi: 10.1186/s13690-024-01418-x (PMC11562729; doi:10.1186/s13690-024-01418-x)
Supplement: Supplementary file 1 — Supplementary Material 1 [file 13690_2024_1418_MOESM1_ESM.pdf]

University of Antwerp – research on abortion trajectories

Dear participant,

To **improve access to abortion care**, the University of Antwerp is trying to map the barriers in the path to an abortion. We would also like to hear whether you have experienced any barriers and therefore ask you to complete a short questionnaire.

The questionnaire takes about **10 minutes** to complete and you can quit at any time. Your participation is completely voluntary and does not affect abortion care. We will link your data to your medical record at the abortion center, but the data will be processed anonymously afterward. More information about the study can be found on the information sheet, attached to this questionnaire.

Are you willing to participate in this study?

- ○ Yes *→ skip to QB*
- ○ No *→ go to QA*

QA It is understandable that you chose not to complete the questionnaire, and we appreciate your honesty. If you wish, you can still help us by indicating why you do not want to participate.

- ○ I don't have time to complete the survey.
- ○ I don't feel like completing the survey.
- ○ I have another reason. Write here:

  ____

- ○ I still want to participate in the survey. → *start completing the questionnaire*

QB What is your date of birth? (dd/mm/yyyy)

/ /

Q1 Do you remember the day your last period started?

- ○ Yes, I know the exact date my last period started.

  Date: / / → *skip to Q2*

- ○ No, I don't know the date exactly, but I have an idea of when that was approximately.

  Estimated date: / / → *skip to Q2*

- ○ No, I don't know. → *go to Q1_A*

Q1_A Can you estimate when you think you had your last menstrual period?

- ○ Less than a month ago.
- ○ Between 1 and 2 months ago.
- ○ Between 2 and 3 months ago.
- ○ Between 3 and 6 months ago.
- ○ Between 6 months and 1 year ago.
- ○ More than a year ago.
- ○ No, I can't assess it.

Q1_B You mentioned that you have not had a menstrual period for a while. **Which of the following applies to you?**
You can select multiple options.

- ☐ My period is very irregular.
- ☐ I don’t have a monthly period due to hormonal contraception.
- ☐ I just gave birth.
- ☐ I am breastfeeding.
- ☐ Another reason: ____

Q2 Do you remember the day you first wondered if you might be pregnant?
This could be the day you first experienced pregnancy symptoms such as a missed period, nausea, fatigue, etc.

○ Yes, I know the exact date.

Date: / /

○ No, I don’t know the date exactly, but I have an idea of when it was.

Estimated date: / /

○ No, I don’t know.

Q3 Do you remember the day you <u>first</u> had a positive pregnancy test?
By a pregnancy test, we mean either a urine test, a blood test or an ultrasound

○ Yes, I know the exact date.

Date: / /

○ No, I don’t know the date exactly, but I have an idea of when it was.

Estimated date: / /

○ No, I don’t know.

○ Not applicable, I didn't do a test yet.

We would also like to understand what causes the trajectory towards abortion care to be possibly delayed or complicated. We will describe some possible reasons or situations at each step in the process. We would like to ask you to indicate which of these apply to you. That way, we can get a better picture of your specific situation

---

Q4 Were there factors that **prevented you from realizing you were pregnant right away?** Indicate what applied to you:

	Yes	No
My period was irregular, therefore, I did not immediately realize I was pregnant.	○	○
I thought I couldn't get pregnant.	○	○ → *don't answer Q4_A*
I did not immediately link the symptoms I was having to a possible pregnancy.	○	○
I had no typical pregnancy symptoms such as nausea, vomiting, or fatigue.	○	○
I (unconsciously) denied the possibility of being pregnant.	○	○

Q4_1 Was there anything else that prevented you from realizing you were pregnant right away? Write it here.

NOTE: Only answer this question if you indicated that you thought you couldn’t get pregnant.

Q4_A You indicated that you thought you couldn't get pregnant. Can you indicate why you thought this?
You can select multiple options.

- ☐ Because I was using contraception.
- ☐ Because I thought I was infertile.
- ☐ Because I had previously undergone fertility treatments to become pregnant.
- ☐ Because I thought I wouldn't get pregnant so easily at my age.
- ☐ Because I didn't realize I could get pregnant from the sexual intercourse I had.
- ☐ Other. Write here:

____

NOTE: Answer this question only if you already taken a pregnancy test. Did you not do one yet? Skip to Q5_GT.

Q5_T Were there factors that made it difficult for you **to take a pregnancy test?**
Indicate what applied to you:

	Yes	No
I didn't know what I would do if I turned out to be pregnant, so I postponed the test.	○	○
I didn't immediately think of the possibility of doing a pregnancy test.	○	○
I was concerned about how my (sex)partner would react to a positive pregnancy test, so I postponed the test.	○	○
I did not immediately know where to obtain a pregnancy test.	○	○
It took me a while to find an appropriate time to take a pregnancy test.	○	○
I found it difficult to raise enough money to pay for a test.	○	○

NOTE: Answer this question only if you have not yet taken a pregnancy test.

Q5_GT Were there factors that made it difficult for you **to take a pregnancy test?**
Indicate what applied to you:

	Yes	No
I didn't know what I would do if I turned out to be pregnant, so I postponed the test.	○	○
I hadn't thought about the possibility of doing a pregnancy test yet.	○	○
I was concerned about how my (sex)partner would react to a positive pregnancy test, so I postponed the test.	○	○
I did not immediately know where to obtain a pregnancy test.	○	○
It took me a while to find an appropriate time to take a pregnancy test.	○	○
I found it difficult to raise enough money to pay for a test.	○	○

Q5_1 Was there anything else that made it difficult for you to take a pregnancy test?
Write it here:

Q5_A Have you visited a doctor or other healthcare provider regarding your current pregnancy before contacting the abortion center?
You can select multiple options:

- ☐ Yes, a general practitioner.
- ☐ Yes, a gynecologist.
- ☐ Yes, a midwife.
- ☐ Yes, another type of healthcare provider.

Write the type of health care worker here:

____

- ☐ No

Q6_X How sure are you at this point that you want an abortion?

Circle the number that best fits your feelings.

I am still in doubt — My decision is firm

1 2 3 4 5 6 7 8 9 10

Q6_Y How difficult was it for you to decide about the abortion?

Circle the number that best fits your feelings.

Not difficult at all — Very difficult

1 2 3 4 5 6 7 8 9 10

Q6 Are there factors that make/made your **decision on abortion** more difficult?
Indicate what applied to you:

	Yes	No
I doubt(ed) for a long time whether an abortion was the right decision.	○	○
My (sex) partner (had) doubts for a long time whether an abortion was the right decision.	○	○
My (sex) partner and I have completely different opinions about the pregnancy.	○	○
I did not intend to have an abortion at first, but due to an unexpected event, I start(ed) doubting.	○	○ *→ don't answer Q6_A*
I wait(ed) to decide because I hoped that the relationship with my (sex)partner would improve.	○	○
I feel/felt ashamed that I am considering/considered having an abortion.	○	○
I am/was disappointed in myself because I am considering/considered having an abortion.	○	○
I feel/felt pressure to make a certain choice.	○	○ *→ don't answer Q6_B*
Abortion is difficult within my religion.	○	○

Q6_1 Was there anything else that made this decision difficult for you?
Write it here:

NOTE: only answer this question if you indicated that the pregnancy might became unwanted due to an unexpected event.

Q6_A You indicated that the pregnancy became unwanted due to an unexpected event. Can you indicate what this event was?
You can select multiple options.

- ☐ I lost my job.
- ☐ The relationship with my partner ended.
- ☐ I learned that the pregnancy would pose risks to my health.
- ☐ During a medical consultation, I learned something about the fetus that led me to decide not to continue the pregnancy.
- ☐ Another event. Write it here: ____

NOTE: Only answer this question if you indicated that you feel pressure to make a certain choice regarding the pregnancy.

Q6_B You mentioned that you feel pressure to make a certain choice. Can you also indicate which choice this is about?

- ◯ I feel pressure to choose abortion.
- ◯ I feel pressure to choose pregnancy preservation.
- ◯ None of the above.
- ◯ I'd rather not answer.

Q7 Were there factors that made it difficult for you to **schedule the first consultation at the abortion center?**

Indicate what applied to you:

	Yes	No
I was afraid to call the abortion clinic.	○	○
I was scared because I didn't know what to expect in terms of the procedure and the associated pain.	○	○
The suggested appointment times didn't immediately work for me (e.g., due to work, childcare, planned travel...).	○	○
It took more than a week to get a first consultation.	○	○
I was referred to another center.	○	○
I made different phone calls first to find out which center could provide a first consultation the soonest.	○	○
It was difficult to find a suitable moment without having to inform someone.	○	○
I needed time to find out where I could obtain an abortion.	○	○

Q7_1 Was there anything else that made it difficult for you to make an appointment?

Write it here:

Q8 Were there factors that made it more difficult for you **to attend the first consultation at the abortion center?**

Indicate what applies to you:

	Yes	No
I found it difficult to gather enough money to pay for the treatment.	○	○
I found it difficult to gather money for transportation to the abortion center.	○	○
Due to unforeseen circumstances, I had to cancel the scheduled first consultation.	○	○
I still had doubts, so I canceled the scheduled first consultation and rescheduled it later.	○	○
I missed the scheduled first consultation and rescheduled it later.	○	○
I had to travel a long distance to come to the abortion center.	○	○
I am not/no longer in compliance with health insurance & found it difficult to get this in order.	○	○
The aid agency paying for me (OCMW, Fedasil, Red Cross) took a long time to give their approval.	○	○

Q8_1 Was there anything else that made it difficult for you to attend the first appointment at the abortion center? Write it here:

Q9_A The following statements are about talking to your close friends and relations about your abortion. Select what best describes your experience.

	Never	Once	More than once	Many times	*I'd rather not answer*
I have had a conversation with someone I am close with about my abortion.	○	○	○	○	○
I was open with someone that I am close with about my feelings about my abortion.	○	○	○	○	○
I felt the support of someone that I am close with.	○	○	○	○	○

Q9_B The following statements are about talking to your close friends and relations about your abortion. Select what best describes your experience.

	Strongly disagree	Disagree	Neither agree nor disagree	Agree	Strongly agree	*I'd rather not answer*
I can talk to the people I am close with about my abortion.	○	○	○	○	○	○
I can trust the people I am close to with information about my abortion.	○	○	○	○	○	○
I feel supported in my decision on abortion by the people I am close with.	○	○	○	○	○	○

Q9_C The following statements are about the things you might worry/worried about when deciding on abortion. Select what best describes your experience.

	Not worried	A little worried	Quite worried	Extremely worried	*I'd rather not answer*
Other people might find out about my abortion.	○	○	○	○	○
My abortion would negatively affect my relationship with someone I love.	○	○	○	○	○
My abortion would negatively affect my relationship with someone I love.	○	○	○	○	○
People would gossip about me.	○	○	○	○	○
People would judge me negatively.	○	○	○	○	○

Q9_D Do you know someone who has had an abortion?

○ Yes, I do.

○ No, I don't

○ *I prefer not to answer.*

Q10_A Regardless of whether you belong to a particular religion, how religious would you say you are? Answers are on an 11-point scale ranging from not at all religious (0) to very religious (10).

Circle the number that best fits your views.

Not at all religious										Very religious
0	1	2	3	4	5	6	7	8	9	10

Q10_B In politics, people talk about 'left-wing' (progressive) and 'right-wing' (conservative). In general, how would you place your views on this scale from left-wing (0) to right-wing (10)?

Circle the number that best fits your views.

Left-wing										Right-wing
0	1	2	3	4	5	6	7	8	9	10

Q11 We would also like to interview some women to hear about their personal experiences with the process of obtaining an abortion. Would you be willing to participate in such an interview?
If yes, you can provide your email address here:

____

Thank you for participating in this questionnaire on abortion care. Your input and experiences are valuable and will contribute to our understanding and improvement of access to abortion care. We greatly appreciate your time and openness. If you have any further questions or comments, please do not hesitate to contact us.
info.ato@uantwerpen.be

Thank you again for your contribution!

INFORMATIONSHEET FOR STUDY PARTICIPANTS

Researcher: Anna Wallays
Institution: University of Antwerp, Department of Sociology

Purpose and description of the study

This study aims to reconstruct abortion trajectories and to identify the barriers encountered within the Flemish context. The study is conducted from the perspective of individuals who request an abortion at one of the Dutch-speaking abortion centres in Belgium. Participation in the research is done through a self-administered smartphone survey. The research takes place within the Department of Sociology at the University of Antwerp. The study is part of the doctoral research of the responsible researcher, Anna Wallays, under the supervision of Prof. Dr. Sarah Van de Velde.

Procedure

You will be asked to complete an online questionnaire about significant moments in your abortion trajectory. Additionally, you will be asked about any barriers you have experienced in your trajectory. The questionnaire will take approximately 10 minutes of your time. It is important that you answer the questions as honestly and accurately as possible.

Risks

There are no known risks associated with completing this questionnaire.

Benefits

The research can contribute to a better understanding of the trajectories women go through to obtain an abortion and the barriers they encounter. The results of the research may potentially lead to improved access to abortion care and related policy decisions.

Confidentiality

The answers you provide will be linked to your patient record at the abortion centre based on your date of birth. This is done to take the complete context into account and to avoid having you answer the same questions multiple times. Once this link has been established, all answers provided by you will be anonymized. The anonymized data will be stored on a secured drive and will only be shared for further research within the research group. You always have the right to modify your answers or withdraw your participation. In that case, your answers will be deleted and not included in the analysis.

Consent

Your participation in this research is entirely voluntary. You can stop completing the questionnaire at any time. Your choice to participate or not will have no impact on the services provided by the abortion centre.

Contact

If you have any questions about this research, you can contact the researchers via email: info.ato@uantwerpen.be or phone: +32 485 75 00 43.
