# Supplementary material for: Abortion Trajectory, Timing, and Access Study (ATTAS): study protocol
Source: Arch Public Health. 2024 Nov 13;82:211. doi: 10.1186/s13690-024-01418-x (PMC11562729; doi:10.1186/s13690-024-01418-x)
Supplement: Supplementary file 2 — Supplementary Material 2 [file 13690_2024_1418_MOESM2_ESM.pdf]

Table 1: Barriers from literature review to final questionnaire

Literature review – initial list of barriers	Fieldwork and FGD – edited list of barriers	Pilot – list of barriers final questionnaire
**LMP - first suspicion of pregnancy (stage 1)**		
- My period is irregular<br>- I thought the bleeding/spotting I had to be menstruation<br>- I had previous experience of stress-related amenorrhea<br>- I'm unsure about when I had my last menstrual period	- My period is irregular	- My period was irregular, therefore, I did not immediately realize I was pregnant.
- I was not initially relating the experienced symptoms to a possible pregnancy<br>- I had lack of pregnancy symptoms (nausea/vomiting, tiredness)	- I was not initially relating the experienced symptoms to a possible pregnancy<br>- I had lack of pregnancy symptoms (nausea/vomiting, tiredness)	- I did not immediately link the symptoms I was having to a possible pregnancy.<br>- I had no typical pregnancy symptoms such as nausea, vomiting, or fatigue.
- I was using contraception<br>- I was on hormonal birth control and didn't let my period come through for a while<br>- I didn't think that what we did would make me pregnant<br>- I believed I couldn't get pregnant/I felt that pregnancy<br>- was unlikely or impossible my particular circumstances<br>- I was convinced that I was infertile<br>- I'm overweight/obese	- I believed I couldn't get pregnant because…<br>- … I was using contraception<br>- ... I didn't think that what we did would make me pregnant<br>- … I was convinced that I was infertile	- I thought I couldn't get pregnant.<br>- Because I was using contraception.<br>- Because I thought I was infertile.<br>- Because I had previously undergone fertility treatments to become pregnant.<br>- Because I thought I wouldn't get pregnant so easily at my age.<br>- Because I didn't realize I could get pregnant from the sexual intercourse I had.<br>- Other…
	- *… I (unconsciously) denied the possibility of being pregnant*	- … I (unconsciously) denied the possibility of being pregnant<br>- *Anything else?*
**first suspicion of pregnancy - confirming pregnancy through test (stage 2)**		
- I suspected I was pregnant but I didn't do anything about it<br>- I wasn't sure what I would do if I were pregnant	- I wasn't sure what I would do if I were pregnant	- I didn't know what I would do if I turned out to be pregnant, so I postponed the test.
- I did not want to see my regular doctor, and it took time for me to find somewhere else to go for a pregnancy test<br>- I experienced denial about the pregnancy		- I didn't immediately think of the possibility of doing a pregnancy test.
- I was worried how my partner would react	- I was worried how my partner would react	- I was concerned about how my (sex)partner would react to a positive pregnancy test, so I postponed the test.
- I had to wait for more than 48 hours for an appointment at my/a doctors surgery for a pregnancy test		- Visited a doctor or other medical health provider before contacting the abortion center?<br>- Yes, a general practitioner.<br>- Yes, a gynecologist.<br>- Yes, a midwife.<br>- Yes, another type of healthcare provider:
- I was worried how my parent(s) would react		
	- *I could not find a suitable time or place to take a test*<br>- *I didn't know how to get a test/take it*<br>- *I had difficulty paying for the test*	- It took me a while to find an appropriate time to take a pregnancy test.<br>- I did not immediately know where to obtain a pregnancy test.<br>- I found it difficult to raise enough money to pay for a test.<br>- *Anything else?*

confirming pregnancy through test - deciding to have an abortion (stage 3)

- I was not sure/uncertain about having the abortion, and it took me a while to make up my mind and ask for one<br>- It was a difficult decision to make, so it took some time to decide<br>- I was worried about what was involved in having an abortion so it took me a while to ask for one	- It was a difficult decision to make, so it took some time to decide	- I doubted for a long time whether an abortion was the right decision.
- I was hoping/waiting to see if my partner would support me in having a baby<br>- I was waiting for my relationship with my husband/partner to change	- I was hoping/waiting for my relationship to improve	- I waited to decide because I hoped that the relationship with my (sex)partner would improve.
- It took time to talk to my partner	- It took time to talk to my partner	- My (sex) partner had doubts for a long time whether an abortion was the right decision.
- I was afraid to tell my husband/partner that I was pregnant	- I was afraid to tell my partner/husband that I was pregnant	- My (sex) partner and I have completely different opinions about the pregnancy.
- Someone I am close to puts pressure on me not to have an abortion<br>- Someone was discouraging me of having an abortion and this caused a delay in making arrangements	- Someone I am close to puts pressure to continue the pregnancy	- I felt pressure to make a certain choice.<br>- I feel pressure to choose abortion.<br>- I feel pressure to choose pregnancy preservation.
- I/we needed to know what sex the baby was before I/we decided whether to continue with the pregnancy<br>- My relationship with my parent(s) broke down/changed<br>- My partner changed his mind about having a baby<br>- My relationship with my partner broke down/changed<br>- Something in my life changed that made a previously desired pregnancy undesired	- I/we needed to know what sex the baby was before I/we decided whether to continue with the pregnancy<br>- My relationship with my partner broke down/changed<br>- My partner changed his mind about having a baby<br>- My relationship with my partner broke down/changed<br>- Something in my life changed that made a previously desired pregnancy undesired	- I did not intend to have an abortion at first, but due to an unexpected event, the pregnancy became unwanted.<br>- I lost my job.<br>- The relationship with my partner ended.<br>- I learned that the pregnancy would pose risks to my health.<br>- During a medical consultation, I learned something about the fetus that led me to decide not to continue the pregnancy.<br>- Another event. Enter here:
- I had religious concerns<br>- It took time to talk to my parent(s)<br>- I was afraid to tell my parents that I was pregnant<br>- I was hoping/waiting to see if my parent(s) would support me in having a baby<br>- I didn't know that I could get an abortion	- I had religious concerns	- Abortion is difficult within my religion.
	- *I had a conflict of conscience because of my own judgement on abortion*<br>- *I felt ashamed that I was thinking about opting for an abortion*<br>- *I had no safety net of people to support me in this process*<br>- *It dawned on me that continuing the pregnancy would not be without risk (both for health, socially and emotionally)*	- I was disappointed in myself because I considered having an abortion.<br>- I felt ashamed that I considered having an abortion.
		- *Anything else?*

deciding to have an abortion - asking for an abortion (stage 4)

- I was ashamed to call/go to the clinic and ask for an abortion
- I was afraid of how the staff would behave towards me
- I had negative perceptions of public sector facilities
- I was afraid of being recognized
- I was scared/afraid and didn't know what to expect in terms of the procedure and anticipated pain
- I was initially referred to other clinics
- It was difficult getting time off from work
- My care-taking responsibilities made it difficult to make arrangements
- I had difficulties in arranging childcare

- I was ashamed to call/go to the clinic and ask for an abortion
- I was afraid of how the staff would behave towards me
- I was afraid of being recognized
- I was scared/afraid and didn't know what to expect in terms of the procedure and anticipated pain
- I was initially referred to other clinics
- It was difficult getting time off from work
- I had difficulties in arranging childcare
- *I called several centers to find out where I could get a consultation the earliest*

Making an appointment for the first consultation

- I was afraid to call the abortion clinic.
- I was scared because I didn't know what to expect in terms of the procedure and anticipated pain.
- I was referred to another center.
- The suggested appointment times didn't immediately work for me (e.g., due to work, childcare, planned travel...).
- I made different phone calls first to find out which center could provide a first consultation the soonest.
- *I needed time to find out where I could obtain an abortion.*
- *It took more than a week to get a first consultation.*
- *It was difficult to find a suitable moment without having to inform someone.*
- *I needed time to find out where I could obtain an abortion.*
- *Anything else?*

- I had difficulties with my insurance
- I was worried about the cost of the abortion
- I had difficulties with paying for the abortion
- I needed time to raise money to have the abortion
- It took time to find/sort out whether I had to pay for the abortion myself
- It took me a while to obtain the money I needed to travel to the clinic for the abortion
- I couldn't find a place to have an abortion near where I live, so I had to arrange for transportation to get here
- I needed to travel long distances to the clinic
- I was feeling sad/depressed
- I had a feeling of guilt
- I thought the pregnancy was much less advanced than it was when I asked for the abortion
- I didn't think that it was important to have the abortion earlier in the pregnancy
- There was confusion about where I should go to have the abortion
- I didn't know where to get an abortion
- Uncertainty regarding service entitlements

- I had difficulties with my insurance
- I had difficulties with paying for the abortion
- It took me a while to obtain the money I needed to travel to the clinic for the abortion
- I needed to travel long distances to the clinic
- I had a feeling of guilt
- I thought the pregnancy was much less advanced than it was when I asked for the abortion
- Lack of information
- *I did not turn up to my initial appointment for the first consultation*
- *I cancelled my appointment for the first consultation*

Getting to abortion centre for first consultation

- I am not/no longer in compliance with my health insurance and found it difficult to get this in order.
- I found it difficult to gather enough money to pay for the treatment.
- I found it difficult to gather money for transportation to the abortion center.
- I had to travel a long distance to come to the abortion center.
- I still had doubts, so I canceled the scheduled first consultation and rescheduled it later.
- I missed the scheduled first consultation and rescheduled it later.
- Due to unforeseen circumstances, I had to cancel the scheduled first consultation.
- *The aid agency paying for me (OCMW, Fedasil, Red Cross) took a long time to give their approval.*
- *Anything else?*
